# Multi-Wavelength Calibration of a Low-Cost High-Range Turbidimeter: Analysis of the Dispersion Regime

**DOI:** 10.1021/acsomega.5c05055

**Published:** 2025-10-29

**Authors:** Laiz R. Ventura, Alexandre E. Santos, Gabriela V. Buraschi, José L. Paralovo, Marcos N. Gallo, Luiz G. Guimarães, Carlos E. Fellows

**Affiliations:** † Departamento de Física, 28110 Instituto Tecnológico de Aeronáutica, São José dos Campos 12228-900, Brazil; ‡ Departmento de Física, Instituto de Ciências Exatas - ICEx, Universidade Federal Fluminense, Campus do Aterrado, Volta Redonda, RJ 27213-45, Brazil; § Programa de Engenharia Oceânica-COPPE, 28125 Universidade Federal Do Rio de Janeiro-UFRJ, Rio de Janeiro 21945-970, Brazil; ∥ Departamento de Química, Instituto de Ciências Exatas - ICEx, Universidade Federal Fluminense, Campus Do Aterrado, Volta Redonda, RJ 27213-415, Brazil

## Abstract

Turbidimetry, a method for assessing fluid clarity by quantifying suspended particle levels, plays an important role in various fields, including environmental surveillance, sediment measurements, water quality management, and diverse industrial sectors. Various optical instruments are commercially available, normally called turbidimeter. However, no generic calibration that can be used to convert the turbidimeter output to NTU is possible. This work presents the development of a low-cost multiwavelength turbidimeter designed for high-range turbidity measurements (0.5–4000 NTU) in visible and near-infrared spectra (500–1000 nm). Using formazin solution as a turbidimetric standard and introducing the *calibration factor* concept, we were able to determine the identification of multiple calibration zones of the developed sensor. For calibration experiments, a portable spectrometer was used to measure the transmitted light, thereby obtaining a spectrum associated with each standard. Then, analysis of the obtained spectra was performed, enabling characterization of the calibration method employed in the study. Considering various wavelengths in the analysis, the results suggest that the present methodology has the potential to develop environmental monitoring practices and water quality control. More specifically, turbidity measurements can be performed in a wide range of NTU and wavelength values, suggesting the feasibility of conducting analyses over an extensive turbidity spectrum.

## Graphical abstract

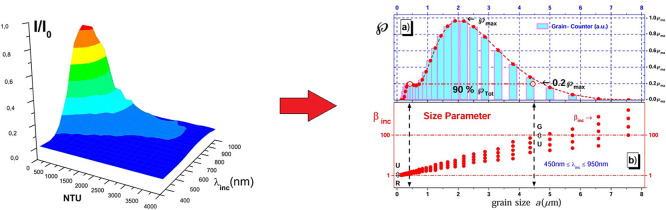

## Introduction

Turbidity is a crucial physical characteristic of fluids, widely employed as a quality parameter in various fields.^1–3^ It quantifies the transparency of the fluid by assessing the presence of both organic and inorganic suspended particles that obstruct the transmission of light through the fluid.^4,5^ For this reason, this type of measurement holds significant importance in environmental monitoring, finding extensive utility in water quality analysis to optimize treatment processes and serving as a valuable tool across various industries. In particular, it plays a crucial role in the food industry, aiding in the detection of potential precipitates in beverages.^6^ Moreover, turbidity measurement techniques have also been found in applications to quantify suspended particles in gases, including smoke or soot as documented in previous studies.^7,8^ Additionally, these methods have been harnessed in renal examinations, contributing to the assessment and quantification of turbidity levels, thereby assisting in the evaluation of renal function and the detection of potential abnormalities, as substantiated by relevant research.^9–11^ Specifically for sedimentation and sediment transport processes in rivers, reservoirs, estuaries, or coastal areas, turbidimeters could give high temporal resolution data for comprehensive knowledge of sediment movement.^12,13^ As has been widely documented in the literature,^14–18^ turbidity is usually measured using modern optical instruments called turbidimeters. These instruments are founded on two fundamental methodologies: The first method, known as turbidimetry, quantifies light transmission at an angle of 0^◦^ to the incident beam, measuring the attenuation of light intensity as it passes through a sample. On the other hand, the second technique, called nephelometry, evaluates the scattering of light, usually at an angle of 90^◦^, to determine the concentration of particles based on the intensity of the scattered light. Both methods are widely used in environmental monitoring (for example, assessing turbidity in natural waters) and industrial process flows (such as optimizing the transparency of liquids in beverage production), offering distinct advantages depending on the optical properties of the sample and the particle load.

In the present study, a low-cost turbidimeter was developed using the turbidimetry methodology. However, some points need to be raised. The first one is that, probably due to instrumentation and facilities limitations, some turbidimetry studies are limited to low concentration values, usually up to 1500 NTU.^19,20^ In addition, it is important to point out that many works are also wavelength band limited, since only a few fixed wavelengths are employed.^21,22^ These operating range restrictions persist even in recent solutions, as evidenced by several studies with complementary limitations. In their apparatus using infrared LEDs as the light source, Sperandio et al.^23^ obtained reliable measurements only in the 100 to 1000 NTU range. Similarly, Putra et al.^24^ demonstrated a loss of precision for values obtained above 300 NTU in their device that uses LEDs in the visible region as a source, reporting RMSE values of 11.87 to 20.63 NTU in their operating range of 0 to 500 NTU. For specialized eutrophication monitoring, Rocher et al.^25^ limited their infrared-based sensor to the 0–200 NTU range in sediment and algae mixtures. On the other hand, Sanchez et al.^26^ developed a low-cost turbidity prototype using infrared LEDs as a light source, capable of measuring low turbidity values, in the 0–50 NTU range. For these reasons and in order to try to develop a more clear physical calibration criterion, we chose to perform our analysis expanding the range of wavelengths to be used in this study, as well as widening the turbidity range of the material analyzed, using turbidity levels up to 4000 NTU. By introducing the calibration factor (γ = 2_π_NTU/λ), a dimensionless parameter that correlates turbidity (NTU) and wavelength (λ), we identified distinct calibration zones across the operational spectrum (500–1000 nm, 0.5–4000 NTU). These zones, categorized by the predominance of specific light scattering regimes (e.g., Rayleigh, Mie, and geometric scattering), allow for adaptive calibration protocols tailored to different turbidity ranges and particle size distributions. This exploration of large concentrations at such an extensive range is relevant for practical applications, as numerous real-world settings and industrial processes often entail significantly higher turbidity levels. Besides that, to perform measurements in a wide range of wavelengths, a spectrometer was used to measure transmitted light, where we obtain a spectrum related to each concentration standard at turbidity levels.

Formazin, a synthetic polymer, is often used to simulate suspended particles in liquids, being crucial to enable an accurate assessment of fluid turbidity. Particularly in turbidimetry, an optically based method widely utilized for turbidity measurement, formazin is extremely important. Through this method, it is possible to quantify the scattering of light caused by particles present in the sample. The correlation established between the intensity of scattered light and the particle concentration allows for a reliable calibration of turbidimeters. For the sake of clarity, we have adopted the term NTU (Nephelometric Turbidity Units) as a direct substitute for formazin concentration throughout this study. The calibration process is aligned with internationally recognized standards, notably ISO 7027 ^27^ and USEPA Method 180.1,^28^ which prescribe formazin as the primary reference material. Although turbidity units vary depending on methodology, FNU (for nephelometry according to ISO 7027) and AU/FAU (for turbidimetry), they exhibit a 1:1 equivalence with NTU (for USEPA Method 180.1 for white light nephelometry) when calibrated with formazin suspensions (1 NTU = 1 FNU = 1 AU = 1 FAU). This numerical parity is recognized in ISO 7027:2016 ^27^ and reinforced by Standard Methods for cross-method equivalence under formazin traceability.^29^ This numerical equivalence ensures compatibility with regulatory frameworks and historical data sets, even though our low-cost turbidimeter operates via transmitted light (turbidimetry) instead of nephelometry. While conventional commercial systems typically adhere to narrow ranges (for example, 0–1000 NTU) and fixed wavelengths (such as 860 nm according to ISO 7027), our methodology extends applicability by analyzing multiple wavelength (500–1000 nm) and high-range turbidity measurements (up to 4000 NTU). Although the development and calibration of low-cost turbidity sensors is a well-studied field, our research advances in this knowledge by introducing three main contributions: (1) the extension of wavelength and turbidity operating ranges beyond the typical limitations found in most devices; (2) the modeling of nonlinear absorption by means of a Padé approximation to describe complex scattering regimes; and (3) the identification of specific scattering mechanisms (Rayleigh, Mie, and geometric) by means of the dimensionless calibration factor γ. This factor allows turbidity to be classified into different operational zones, establishing an adaptable calibration protocol for different scenarios. This development provides a robust physical framework for dealing with real-world challenges, such as industrial effluents and sediment-laden waters, where turbidity often exceeds standardized limits.

The manuscript is structured as follows: In the Materials and Methods section, we first examine the instrumental design and operational principles of the low-cost turbidimeter, followed by a size distribution analysis of formazin and their implications for light scattering dynamics in the Results and Discussion section. This empirical framework allows the derivation of wavelength and concentration dependent turbidity profiles, culminating in a robust calibration protocol based on light scattering theory. Finally, the conclusion summarizes the main findings, emphasizing the methodological advances and their practical relevance for the quantification of turbidity in various analytical contexts.

## Materials and Methods

### Experimental Setup

In the following sections, the measurement process will be presented, which encompasses the synthesis of formazin, the creation of our custom turbidimeter and the transmittance measurements. The measurements were conducted within the wavelength range of 500 to 1000 nm. To fully meet the requirements of this study, the creation of formazin standards has become a fundamental and indispensable step. These formazin standards play a central role in enabling the calibration and verification of turbidity measurement instruments, notably turbidimeters. Thus, it was necessary to produce formazin standards, essential for the calibration of the measurement instrument in question. The concentration values of the formazin standards obtained are presented in Table 1. The manufacturing process of these standards involved the use of two chemical compounds, hydrazine sulfate with a purity of 99%, and hexamethylenetetramine, also with a purity of 99%. Both compounds were supplied and certified by ACS Científica.^30,31^ The initial procedure consisted of creating the 4000 NTU standard. This was achieved through a carefully calculated dilution, incorporating 1% w/v of hydrazine sulfate in 50 cm^3^ of distilled water and 10% w/v of hexamethylene tetramine also in 50 cm^3^ of distilled water. After the dilution, both compounds were mixed in a single container. Subsequently, after a period of 48 h, the standard reached its complete formation and stabilization. The remaining concentrations were obtained through progressive dilutions of the initial 4000 NTU standard. Thus, this dilution process resulted in the generation of a diverse set of 20 standards, each representing different concentration levels. The standards were used immediately after preparation to generate calibration curves for the instrument used in our investigation.

**Table 1:** Turbidity Values (in NTU) of Formazin Standard Solutions

Sample name	NTU value
Standard 1	0.5
Standard 2	2.5
Standard 3	5
Standard 4	10
Standard 5	25
Standard 6	50
Standard 7	100
Standard 8	300
Standard 9	500
Standard 10	700
Standard 11	1000
Standard 12	1500
Standard 13	1800
Standard 14	2000
Standard 15	2100
Standard 16	2300
Standard 17	2500
Standard 18	3000
Standard 19	3500
Standard 20	4000

#### The Turbidimeter

In this study, our objective was to improve the measurement techniques by developing a low-cost turbidimeter. This device is visually depicted in Figure 1a, while a detailed view of its assembly process is provided in Figure 1b, which has been divided into intuitive stages represented from left to right. The technical drawings of the developed device are available in the Supporting Information. In the initial stages, we introduced the tungsten lamp, accompanied by its crucial securing bracket, which plays an integral role in maintaining stability within the sensor body. As the assembly process unfolds, we come upon the sample compartment, a pivotal element in the system. Here, formazin standards are introduced through purposefully designed apertures within the sensor body. The sample compartment is completely isolated from the rest of the turbidimeter body by fused quartz windows, allowing samples to be introduced either in flow or in a static manner. This step plays a crucial role in the calibration and referencing of turbidity measurements. The introduction of formazin into the sample compartment establishes a reliable foundation for evaluating the turbidimeter’s ability to detect various levels of dispersion. The subsequent component is the compartment that houses the optical fiber for spectrum measurement. The optical fiber used was made of quartz, with a core diameter of 400 μm and a length of 2 m. Connecting the turbidimeter to an optical fiber allows the advantage of using the device in a fixed position, with intensity measurements taken remotely. The cylindrical and movable design of this compartment is of significant importance, as it offers flexibility to adjust the optical path l . This capability enables the alteration of the sample volume, thus directly impacting the observed dispersion. The ability to adjust the length of the optical path, ranging from 6 mm to 30 mm, offers versatility to the device, allowing it to adapt to a variety of measurement scenarios. Specifically, in our experimental setup, for any incident wavelength and assuming the formazin as poor electrical conductor material,^32^ we also assume an effective penetration length value similar to the optical path l≈14mm .^33^ Besides, it is important to emphasize that the choice to manufacture the entire piece using aluminum presents significant advantages. Aluminum, renowned for its durability and malleability, not only eases the manufacturing process but also contributes to the turbidimeter’s robustness and longevity.

**Figure 1:**
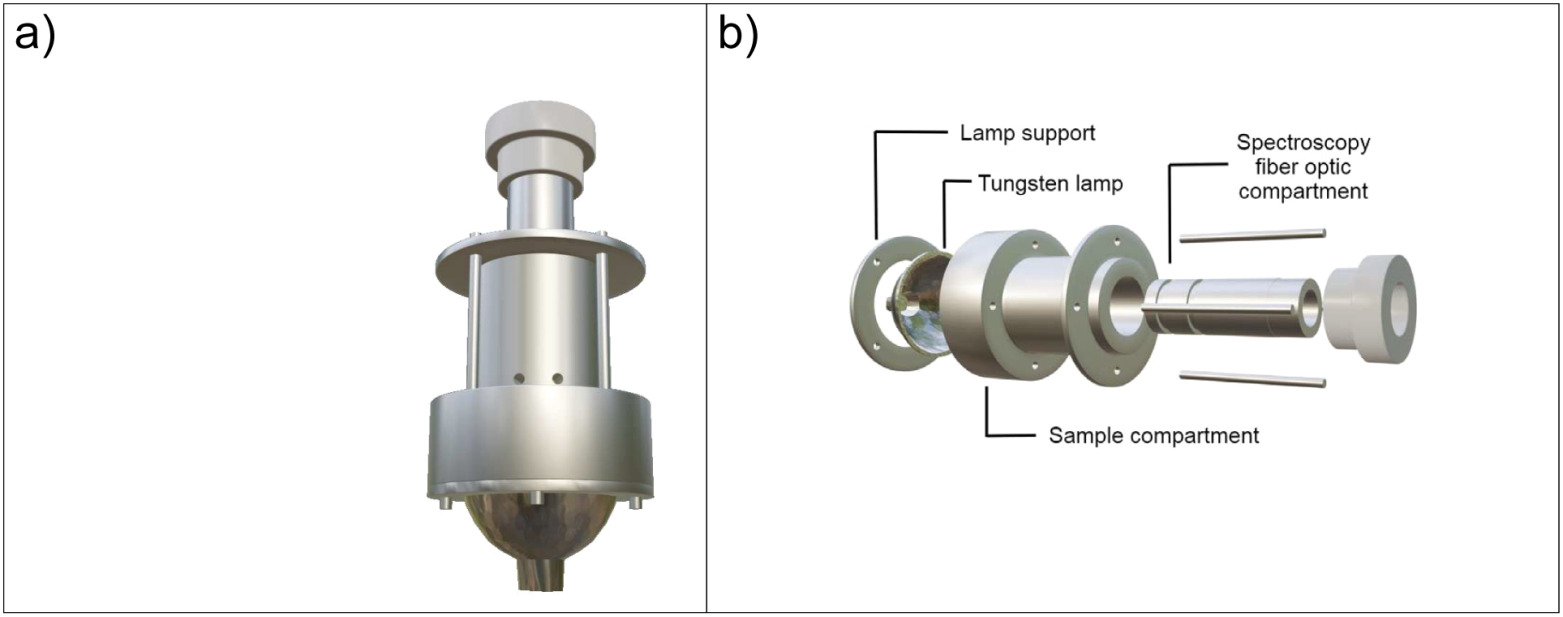
Schematic of the experimental setup. It is presented in panel (a) the representation of the developed turbidimeter, while detailed view of its assembly process is shown in panel (b).

#### Spectral Transmittance Measurements

Transmittance measurements were conducted by directly coupling an optical fiber to the optical fiber compartment. The opposite side of the fiber was connected to an Ocean Optics Red Tide USB650 mini Spectrometer,^34^ which has the capability to record wavelengths ranging from 350 to 1100 nm, with resolution of 2 nm. The advantage of using this type of spectrometer comes directly from its multiplexing capability, which allows measurements to be made at all wavelengths simultaneously. All measurements were performed with an integration time of 100 ms, and 10 averages were collected for each measurement to ensure the acquisition of robust statistical data. The standards used in the experiment are detailed in Table 1 shows the normalized spectrum acquired for 20 standards, with the reference spectrum for distilled water at 0 NTU subtracted from the range of 500 to 1000 nm. During this procedure, it was possible that the spectral normalization was carried out based on the maximum count amplitude of the spectrometer.

## Results and Discussion

### Light Scattering and Particle Size Effects

In this subsection, some size effects on light scattering by formazin are discussed. To do this, it is essential to analyze the percentage size distribution P of the formazin grains. Specifically, the particle size of the formazin sample adopted in the calibration tests was also characterized using the Malvern Mastersizer 2000E analyzer (M2000E, Malvern Panalytical.^35^ The M2000E has been designed to measure P , the particle size distributions (PSD) of different sizes in a sample and uses dual wavelength blue (short) and red (long) light and has 52 detectors from 0.01 to 1.000 μm on a single lens. The Mastersizer 2000 applies the Mie theory,^16,36–41^ which solves the equations for the interaction of light with matter. This allows accurate results over a large size range. In the experiment, we used the M2000E with the wet unit, Hydro 2000MU sampler. The dispersion sample (formazin in water) was transferred to the Hydro 2000MU to the recommended obscuration limits (10 to 20%). The agitation was set to 1000 units and the pump to 2500 units on the Hydro 2000MU. The sample was measured in three runs, and the average was used to calculate the average PSD. The PSD is expressed as a percent of volume (see the specific percentage values for P in Figure 2 and Table 2), equivalent to weight percentage, assuming a uniform specific particle density across all particle sizes when calculating the PSD in the Malvern software. The M2000E was configured using standard parameters: a particle refractive index of 1.52, a particle absorption index of 0.1 and a dispersant refractive index of 1.33. In other words, based on Mie theory for light scattering for spheres,^16,36–41^ these granulometric analysis results are given as histograms that represent the distribution of related percent grains P as the particle radius size *a* (in μm) varies. For instance, the top panel in Figure 2a shows that the 90% of total formazin polpulation $P_{\mathrm{tot}}\left(\%\right)$ are in the particle size range 0.38 μm < *a* ≤ μm < *a* ≤ 4.47 μm. Besides, Figure 2a shows also that the maximum population of formazin radius $P_{\max}$ is reached around *a* ≈ 2 μm, while formazin with radius greater than *a* > 4.47 μm or less than *a* < 0.37 μm represents only 8.47% and 1.13% of the population P , respectively.

**Table 2:** Summary of the Main Aspects of Light Scattering by Formazin Grains, Based on Formazin Grains’ Size Distribution^35^ Applied to Mie Scattering Theory^37–41^

Scattering	*a* (μm)	β_inc_ ≡ 2πa/λ_inc_	λ_inc_ (nm)	$P_{Tot}$ (%)
(R)ayleigh	0.13 < *a* ≤ 0.38	β_inc_ ≤ 1	773 ≥ λ_inc_ ≥ 625	1.13%
(U)ndulatory	0.38 < *a* ≤ 4.47	1 < *β*_inc_ ≤ 100	625 >λ_inc_ ≥ 425	**90.40%**
(G)eometric	*a* > 4.47	*β*_inc_ >100	λ_inc_ < 425	8.47%

**Figure 2:**
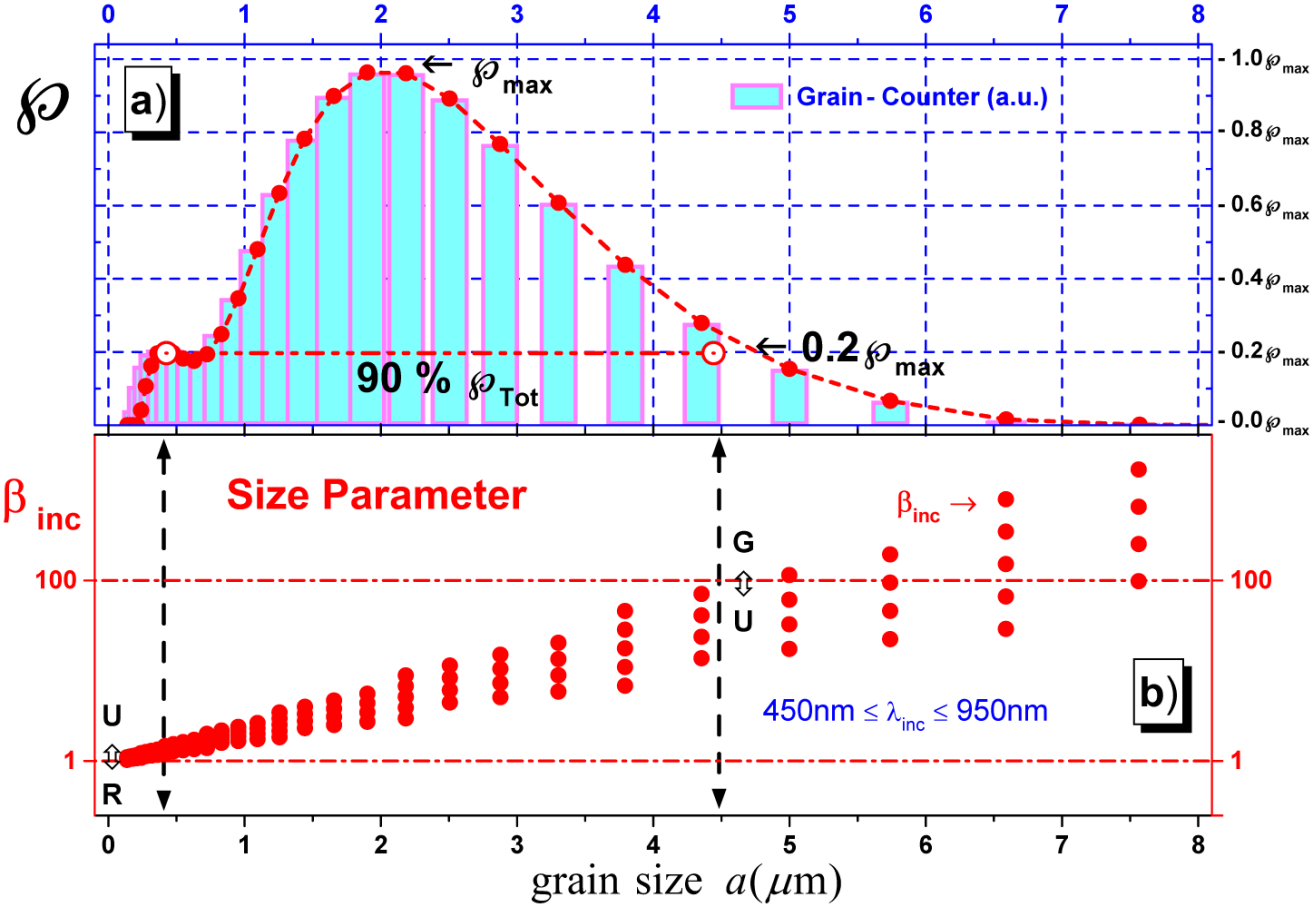
Top panel (a) shows a bar graph corresponding to formazin grains population P^35^ as a function of grain radius *a* (the dashed-dotted line). It can be seen that 90% of the grains have radii in the range 0.38 μm < *a* ≤ 4.47 μm and the grain population reaches a maximum value $P_{\max}$ at *a* ≈ 2 μm. On the other hand, for light wavelengths in range 400 nm ≤ λ_inc_ ≤ 950 nm and based on the Mie scattering theory,^37–41^ the bottom panel (b) shows as, scattered graph, the size parameter (filled circles) β_inc_ behavior as the formazin grain radius *a* varies. It is important to note that under these circumstances, the undulatory light scattering effects are the most important.^37–41^

### Calibration Factor and Nonlinear Response Analysis

Since we have some information on the concentration of formazin, the grain distribution P , the radius of the particle *a* and the wavelength range of the incident light, using the Beer–Lambert formula (BLF), it is possible to infer some characteristics of the light scattering by the formazin solution. If light penetrates a length l through the formazin sample with a given concentration (in NTU), then the light intensity *I* should decay according to BLF,^38–41^ that is

1 $$I\left(\lambda_{\mathrm{inc}},\mathrm{NTU},l\right)=I_{0}\;\exp\;\left[-A\left(\lambda_{\mathrm{inc}},\mathrm{NTU},l\right)\right]$$

Here, based on the present experimental setup, we assume l≈14mm , as well as defining *I*_0_ and the function $A\left(\lambda_{\mathrm{inc}},\mathrm{NTU},l\right)$ as the initial light intensity and absorbance, respectively.^38–41^ On the other hand, in Mie scattering, the absorbance *A* depends on the incident wavelength λ_inc_ through the impact parameter β_inc_, being the impact parameter given by the following the relationship between the radius *a* and λ_inc_:^37–41^

2 $$\beta_{\mathrm{inc}}\equiv \frac{2\pi a}{\lambda_{\mathrm{inc}}}$$

Moreover, as shown in the horizontal axes of the top (a) and bottom (b) of Figure 2, the formazin samples exhibit particle sizes ranging from 0.13 μm ≤ *a* ≤ 4.47 μm, while the wavelengths of the incident light can assume values in the range of 400 nm ≤ λ_inc_ ≤ 950 nm. Applying eq 2, the scatter plot in Figure 2b reveals a wide variation in the size parameter β_inc_, which spans orders of magnitude—from small values (β_inc_ ≤ 1) to large magnitudes (β_inc_ ≥ 100). On the other hand, the literature^37,40,41^ shows that the scattered light for β_inc_ in these asymptotics limits satisfies the physical criteria related to Rayleigh (R) and Geometric (G) scattering, respectively, while formazin with intermediate sizes in the range 0.38 μm < *a* ≤ 4.47 μm, should satisfy the Undulatory (U) light scattering criteria.^37–39,42–44^ In general, the absorbance *A* can also be rewritten in terms of the scattering extinction cross section function $\sigma_{\mathrm{ext}}\left(\beta_{\mathrm{inc}}\right)$ . In Mie scattering, σ_ext_ is given by a series of partial waves.^37–39,42–44^ This series reduces to simple analytical formulas in the asymptotic limits related to R and G scattering.^38,39^ However, the R and G regimes contribute minimally to the present analysis of experimental data. This restriction occurs because, at the R and G asymptotic limits, the size range of the formazin grain populations corresponds to only 1.13% and 8.47% of the total grain population $P_{\mathrm{tot}}\left(\%\right)$ , respectively (see Figure 2a). In addition, we can also see in Figure 2a that 90.40% of the total population of formazin grains $P_{\mathrm{tot}}\left(\%\right)$ has a grain size *a* related to the values of β_inc_ at which the main dispersion characteristics U can occur (see Figure 2b). Finally, in Table 2 summaries the main features discussed above related to light scattering by formazin grains.

However, unfortunately due to many physical complex behavior phenomena related to U scattering, such as wave diffraction, caustics, resonances and light tunneling,^37–39^ it becomes extremely difficult to obtain simple explicit formulas that well describe the σ_ext_ behavior for all present wavelength spectra.^37,42–44^ Then, we make efforts to analyze absorbance *A* behavior based on a more phenomenological picture. In this sense, in Figure 3 is shown the behavior of the relation between the intensities *I* and *I*_0_ as a function of the incident wavelength λ_inc_ and the formazin concentration NTU. In this surface graph (Figure 3), we can notice that the intensity *I* decreases almost exponentially as the NTU concentration increases, but we can see a more complex *I* behavior as λ_inc_ varies. This complex behavior is better observed in the scatter plots shown in Figure 4a and b. In addition, as shown before in Figure 3, we can also see in Figure 4a an almost exponential decay in the normalized intensity *I*/*I*_0_ as the concentration (in NTU) increases. However, Figure 4b shows a pronounced peak in *I*/*I*_0_ for wavelengths around 750 nm, but the same figure also shows very small values of *I*/*I*_0_ over almost the entire incident wavelength range presented here. In other words, a more detailed analysis of Figure 4b shows that there is no trivial behavior of the absorbance *A* as a simple function of the incident wavelength λ_inc_. To try to better understand the complexity of light scattering by suspension of formazin grains, as well as to gain a little more physical insight into this phenomenon, we introduced the *calibration factor γ* (NTU/nm units) which correlates the formazin concentration with the incident wavelength:

3 $$\gamma \left(\lambda_{\mathrm{inc}},\mathrm{NTU}\right)\equiv \frac{2\pi \mathrm{NTU}}{\lambda_{\mathrm{inc}}}$$

**Figure 3:**
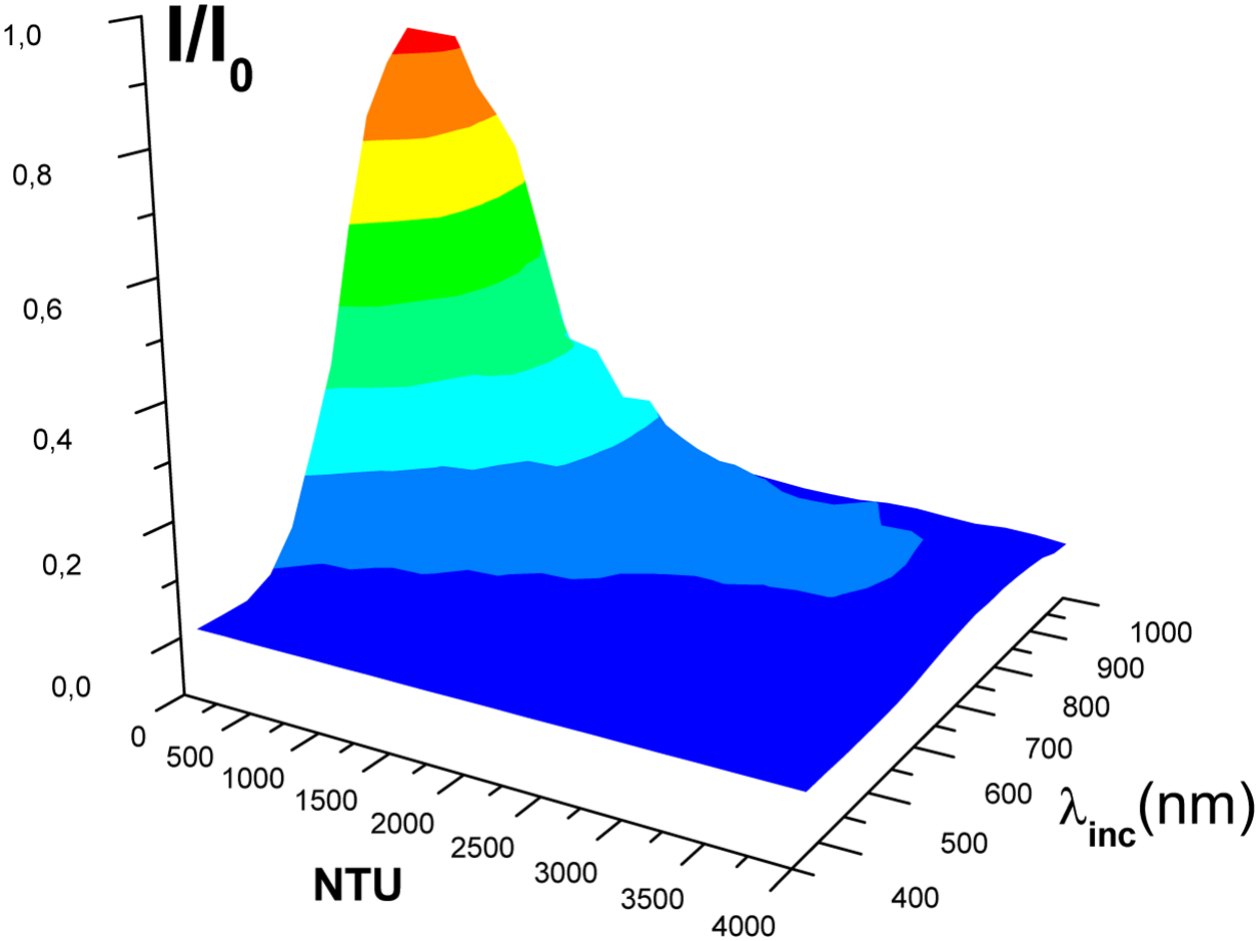
Surface graph—normalized transmitted intensity *I*/*I*_0_ as a function of the formazin concentration (NTU), for 20 standard solutions (see Table 1), and the wavelength of the incident light (λ_inc_ (nm)).

**Figure 4:**
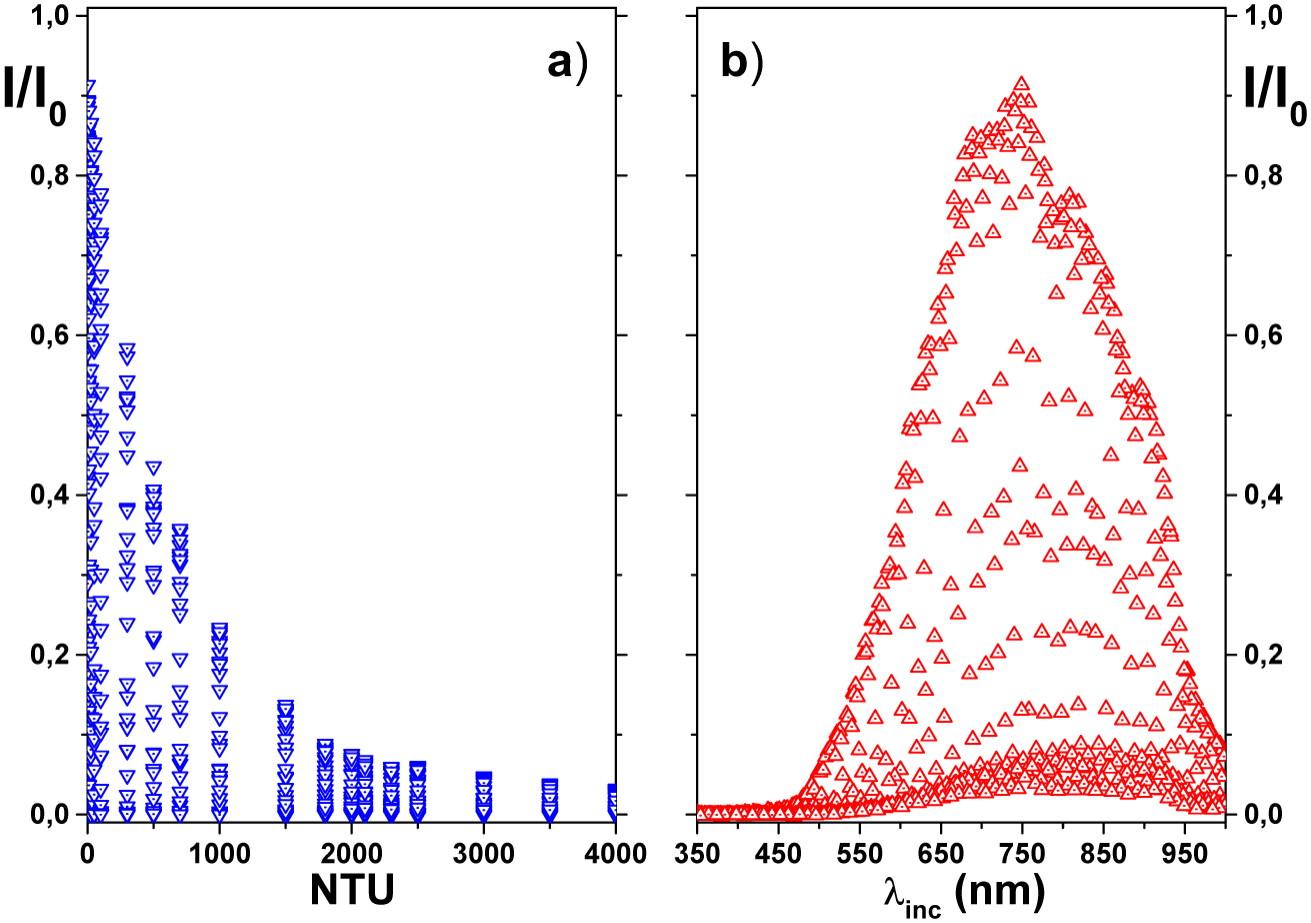
Normalized intensity *I*/*I*_0_ behaves as the concentration of formazin (NTU) (panel a) and as the wavelength of the incident light (λ_inc_ (nm)) (panel b).

To analyze the nonlinear behavior of the scattered intensity *I* as a function of the calibration factor γ, we show in Figure 5 the inverse of the normalized intensity. More specifically, assuming six different values for the incident wavelength (in nm) λ_inc_ = {530, 590, 630, 780, 850, 950}, as well as using all values of formazin concentration standard values adopted in Table 1, we plot the ratio *I*_0_/*I* as a function of γ as scattered data. We notice in Figure 5 that the slow variation in intensity occurs when γ ≪ 1 and the related absorbance *A* is weak, while the opposite situation occurs for γ ≫ 1, where the absorbance *A* reaches high values and *I*_0_ ≫ *I*. In order to better quantify these asymptotic results, first based on the law of BLF ([Disp-formula eq1]) and using the chi-square fitting method,^45^ we adjust the absorbance *A* ≈ *A*[_*L*_] as a straight line with $\chi_{L}^{2}\approx 0.9206$ , namely:

4 $$A_{[L]}\left(\gamma \right)=\pi \frac{926716}{1295472261}\left(1+\frac{\gamma }{Q}\right)$$

with the coefficient *Q* in NTU/nm units given by

5 $$Q=\frac{2740441}{138836392}$$

**Figure 5:**
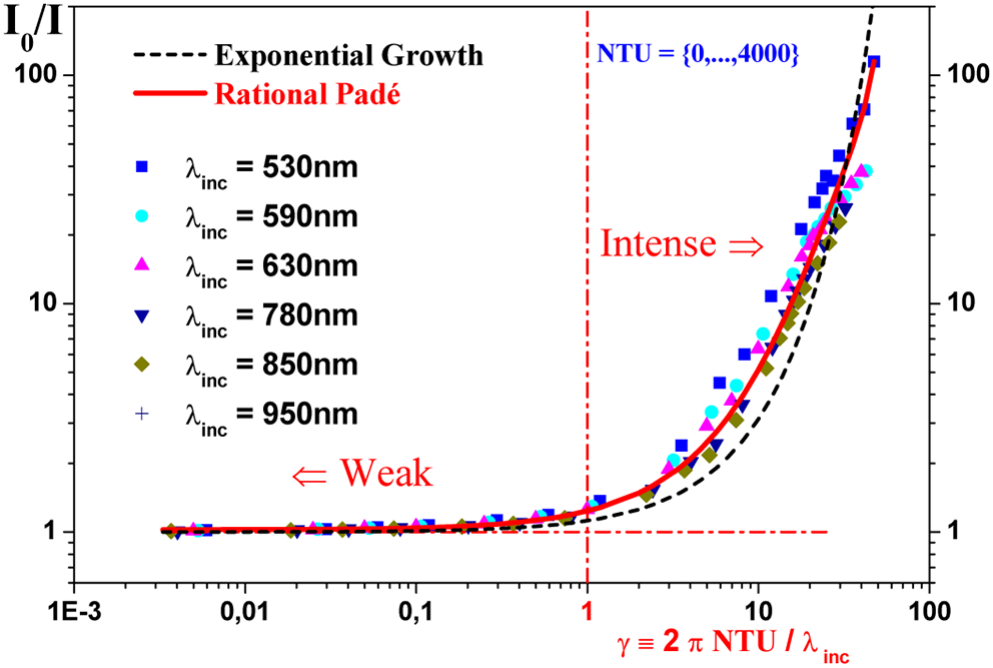
Scatter plots in logarithmic scales log × log to analyze the behavior of the ratio *I*_0_/*I* for six distinct incident wavelengths (λ_inc_, in nm) and formazin concentrations (in NTU), as listed in Table 1, while varying the *calibration factor γ* (see eq 3). Derived from the Beer–Lambert law (BLF, eq 1), the dashed and solid lines correspond to the linear approximation *A*_[*L*]_ ([Disp-formula eq4]) and the rational Padé approximation *A*_[*R*]_ ([Disp-formula eq6]), respectively. These models describe the absorbance *A* ([Disp-formula eq1]) as a function of γ, with the Padé approximation capturing nonlinear effects more effectively across broader γ ranges.

In Figure 5, the above approximation ([Disp-formula eq4]) is shown as dashed lines, but seems to fit the data well only in the weak absorbance region *A* around γ ≤ 1. Then, trying to improve our data analysis, we propose a new approximation for the absorbance *A* ≈ *A*_[*R*]_, where *A*_[*R*]_ is the following Rational Padé polynomial as^45^

6 $$A_{[R]\;}\left(\gamma \right)=\pi \frac{2741258}{369800283}\left(1+\frac{\gamma }{L_{1}}-\frac{\gamma^{2}}{{L_{2}}^{2}}\right){\left(1+\frac{\gamma }{M_{1}}-\frac{\gamma^{2}}{{M_{2}}^{2}}-\frac{\gamma^{3}}{{M_{3}}^{3}}\right)}^{-1}$$

being in this case $\chi_{R}^{2}\approx 0.9776$ and the coefficients *L*_*j*_, *j* = 1, 2 and *M*_*K*_, *K* = 1, 2, 3 (in NTU/nm units) given respectively by,

7 $$\begin{array}{lll} L_{1}=\frac{40299767}{331728737}, & L_{2}=\frac{27407014}{9850485},\\ M_{1}=\frac{830653391}{1281154}, & M_{2}=\frac{292957351}{2939377}, & M_{3}=\frac{146202235}{2176334} \end{array}$$

### Turbidity Intensity Classification and Operational Zones

Furthermore, it is important to notice that for γ > 1, Figure 5 suggests that it is appropriate to adopt the rational Padé approximation above *A*_[*R*]_, since the rational eq 6 seems to fit the data better than the exponential growth based on the linear behavior of the absorbance *A*_[_ *_L_*_]_ ([Disp-formula eq4]). In addition, the logarithmic vertical scale for *I*_0_/*I* in Figure 5 also suggests that we can associate weak turbidity with regions where γ is less than 1, since for 0 < γ < 1 the ratio *I*_0_/*I* ≈ 1 and its slope are almost constant in these regions. However, Figure 5 also shows that, in the opposite case, where γ is much greater than 1, it is possible that there are regions where the occurrence of intense turbidity is reached quickly, since in this asymptotic limit where γ ≫ 1, the slope of the ratio *I*_0_/*I* also varies extremely quickly. In other words, Figure 5 suggests that the strong nonlinear behavior of *I*_0_/*I* as the calibration factor γ varies also motivates us to analyze in more details the slope of *I*_0_/*I* curve as a function of γ. To this end, based on the Padé rational polynomial approximation eq 6, we show in Figure 6 some results related to the asymptotic behavior of the *I*_0_/*I* curve as the calibration factor γ varies. More specifically, the panels in Figure 6a–c) (on a logarithmic horizontal scale) show, respectively, the behavior of the *I*_0_/*I* ratio, its θ slope and the θ′ function, which is the first derivative of the θ slope with respect to γ. For example, the bottom panel of Figure 6a shows that, as the ratio *I*_0_/*I* (γ) reaches the values *I*_0_/*I* (γ_1_) ≈ 4,*I*_0_/*I*(γ_2_) ≈ 40 and *I*_0_/*I* (γ_3_) ≈ 80, the turbidity can be considered weak (or moderate), intense and extreme, respectively. We will see further in the middle panel of Figure 6b that the above criterion for classifying the intensity of turbidity is related to the situation in which the following specific values of the calibration factor γ_1_, γ_2_ and γ_3_ are inflection points of the slope curve θ. More specifically, the top panel of Figure 6c shows that θ′, the first derivative of the slope curve θ, reaches its first maximum at γ_1_ = 8.4, its minimum at γ_2_ = 31.5 and its last maximum value at γ_3_ = 43.3. So, looking again at the panel in Figure 6b, we notice that γ_1_, γ_2_ and γ_3_ are in fact inflection points of θ­(γ), the slope curve of *I*_0_/*I*(γ). On the other hand, the panel in Figure 6b shows also that θ_1_(γ_1_) = 30.30° < θ_2_(γ_2_) = 68.10° < θ_3_(γ) = 80.30°, so the slope curve θ increases as γ increases, and it follows that from the inflection point γ_3_, the slope curve θ­(γ) increases asymptotically up to the maximum value θ_max_(γ_max_)= 90° for γ_3_ < γ_max_ ≈ 60, so for γ around γ_max_ the turbidity intensity is extremely high and bottom panel Figure 6a as well as Figure 5 suggests that in this asymptotic limit the ratio *I*_0_/*I* → ∞. In summary, with these ideas in mind, the concept of calibration factor γ allows us to classify the Turbidity Intensity Ranging (TIR) R*j* as follows:

8 $$\mathrm{TIR}:\left\{ \begin{array}{lll} R1)\mathrm{Weak}\;\mathrm{to}\;\mathrm{Moderate}; & 0<\gamma \leq \gamma_{1}; & 1<I_{0}/I\leq 4\\ R2)\mathrm{Intense}; & \gamma_{1}<\gamma \leq \gamma_{2}; & 4<I_{0}/I\leq 40\\ R3)\mathrm{Extreme}; & \gamma_{2}<\gamma \leq \gamma_{3}; & 40<I_{0}/I\leq 80\\ R4)\mathrm{Extremely}\;\mathrm{High}; & \gamma_{3}<\gamma \leq \gamma_{\max}; & I_{0}/I>80 \end{array}\right.$$

**Figure 6:**
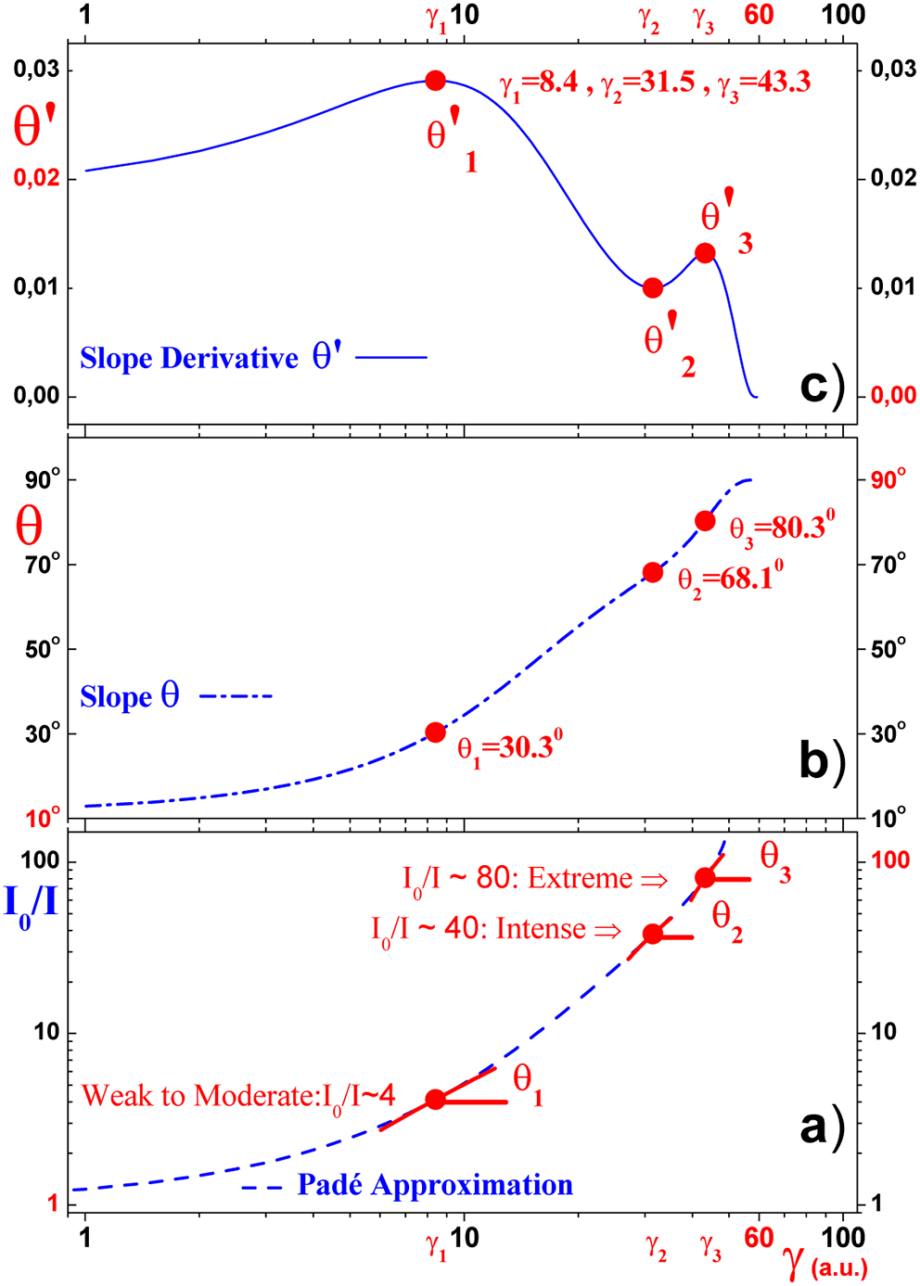
Based on Padé’s polynomial approximation for absorbance *A*_[*R*]_ ([Disp-formula eq6]), panels (a), (b) and (c) show the behavior of the *I*_0_/*I* curve, its slope θ and θ′ first derivative of the slope as functions of γ, respectively. It can be seen that the intensity of the turbidity can be classified as weak or moderate, intense or extreme (see log × log panel (a)) according to the values of the inflection points θ (see panel (b)) or critical points θ′ (see panel (c)) γ_1_ = 8.4, γ_2_ = 31.5 and γ_3_ = 43.3, respectively. It can also be seen in panel (b) that, for γ_max_ ≈ 60 , the slope θ approaches 90° and the turbidity reaches its extreme asymptotic value.

The classification into multiple regions (R1–R4) shown in Figure 7 is a central aspect of this study, as it offers an understanding of turbidity dynamics. Each of these regions represents a distinct rate of turbidity growth, allowing us to identify critical points in the system’s behavior. These demarcations are fundamental as they correspond directly to the calibration ranges of our prototype, establishing the limits where turbidity measurement can be carried out with precision and reliability.

**Figure 7:**
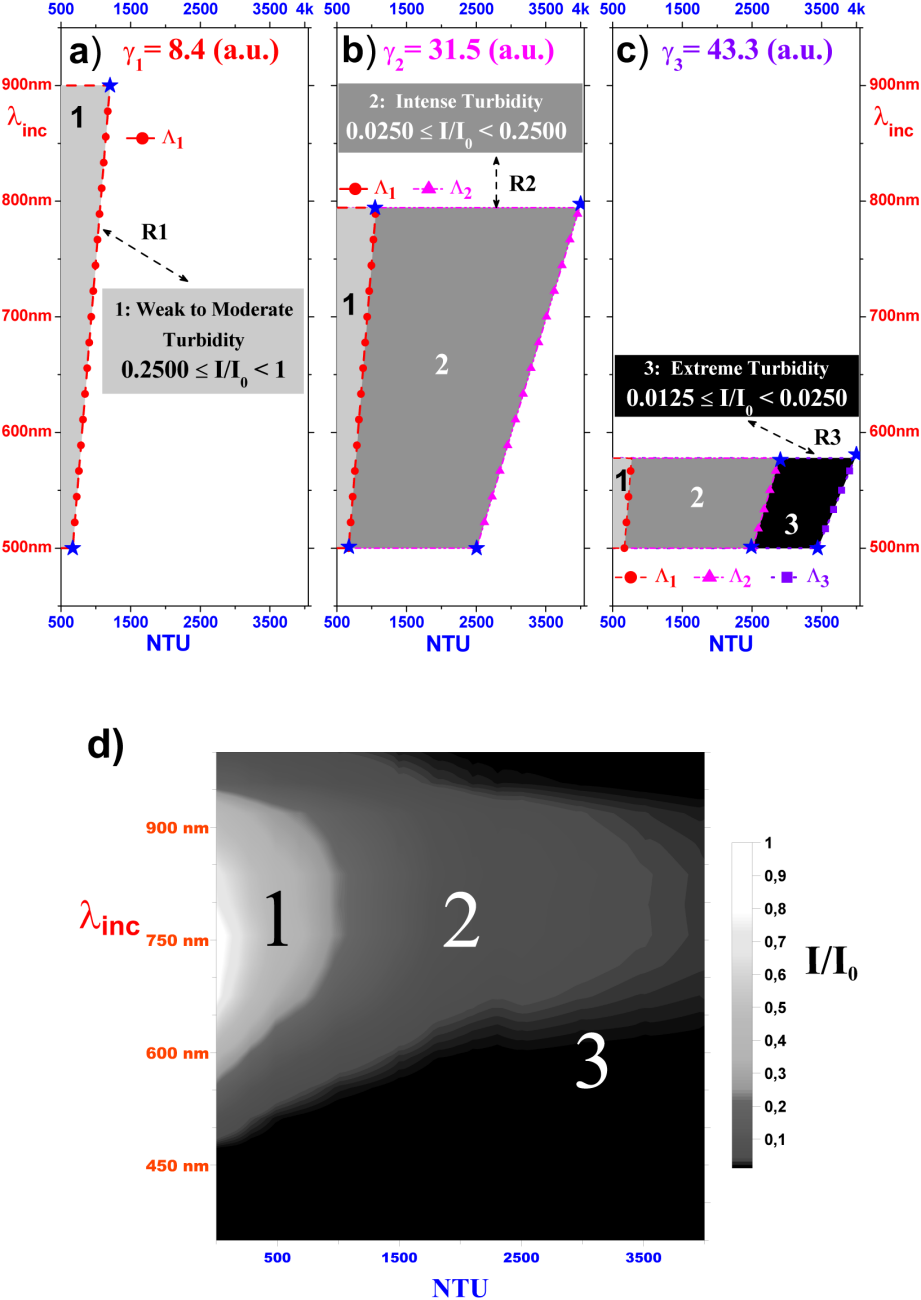
Based on the values of the critical calibration factor γ_*j*_ (see Figure 6), grayscale sketches, the allowed regions in the domain (NTU, λ_inc_) for the turbidity intensity (see eqs 8 and 11), for example, in panels (a–c) the light gray, gray and black regions represent, respectively, the limits of turbidity intensity ranging from weak to moderate in R1, intense in R2 and extreme in R3. In addition, panel (d), also in the (NTU, λ_inc_) domain, shows the allowed turbidity intensity regions R1, R2, and R3 as a grayscale map.

However, from an experimental perspective, it is now necessary to reinterpret the results of eq 8 above, regarding TIR, in their sets original physical parameters, which are the incident wavelengths Λ_inc_ (nm) and *K*_*j*_ the concentrations of formazin in NTU. More specifically, in eq 8, for any and all turbidity intensity ranges R*j* (*j* = 1, 2, and 3), we will be able to map the domain of a given critical calibration factor γ_*j*_ onto the allowed domains of incident wavelength subsets Λ_*j*_ that correlate concomitantly with the concentration subsets *K*_*j*_, respectively:

9 $$\left\{\Lambda_{j}\right\}\subset \left\{\lambda_{\mathrm{inc}}\right\}=\left\{500\;\mathrm{nm},...,1000\;\mathrm{nm}\right\}$$

and

10 $$\left\{K_{j}\right\}\subset \left\{500\;\mathrm{NTU},\cdot \cdot \cdot ,4000\;\mathrm{NTU}\right\}$$

Then, we can define the allowed (*K*_*j*_,Λ_*j*_) domain boundaries of the mapping γ*_j_ →* (*K*_*j*_,Λ_*j*_), simply by rewriting eq 3 as the following line segment:

11 $$\gamma_{j}\to \left(K_{j},\Lambda_{j}\right)\forall :\Lambda_{j}=\frac{2\pi }{\gamma_{j}}K_{j},,\mathrm{with}\;j=\left\{1,2,3\right\}$$

In order to better illustrate the framework described above, we show the related results in Figure 7 as a gray colored schema. For this task, we assume a mixed color criterion in which the light gray and gray colors are just the white color (*w*) superposed on the black color (*b*) in the proportions 1*b*/7*w* and 3*b*/7*w*, respectively. More specifically, the upper panels of Figure 7a–c show a polygonal behavior of the allowed domain (*K*_*j*_,Λ_*j*_), where this situation occurs for the three critical calibration factors γ_*j*_ (*j* = 1, 2, and 3). For example, the panel in Figure 7a shows that R1 (the hatched area in light gray with vertices represented by filled stars ★) is the polygon externally bounded by the line segment Λ_1_ (see the dashed lines). Similarly, it can be seen in the panels of Figure 7b and c that the regions of allowed turbidity intensity R2 and R3 (hatched areas in gray and black, respectively) have external boundaries limited by the line segments Λ_2_ and Λ_3_, respectively. It is also important to note in the panels of Figure 7a–c that, depending on the concentration value *K*_*j*_, a given incident wavelength value Λ_*j*_ can simultaneously be part of different turbidity intensity domains R*j*, for example, as shown in the panel of Figure 7c, incident wavelengths in the range 500 nm ≤ Λ_*j*_ < 600 nm (*j* = 1, 2, and 3) are allowed in the three intensity domains R1, R2, and R3. Finally, the bottom panel of Figure 7d is a grayscale color map of the normalized intensity *I/I*_0_ as a function of the incident wavelength λ_inc_ (in nm) and the formazin concentration (in NTU). Notice that, unlike the case of Figures 3 and 4, now based on the concept of the calibration factor γ ([Disp-formula eq3]) and the TIR ([Disp-formula eq8]), the panel Figure 7d as a gray color map sketching points in the allowed intensity domains 1, 2, and 3 of R*j*, give us some physical insights into the fascinating features analyzed above of light scattering by formazin grains.

## Conclusions

In this introductory work, a low-cost turbidimeter was developed (see Figure 1). This device perform analysis of light scattering by formazin particles across a broad concentration range (0.5–4000 NTU) and multiple wavelengths (500–1000 nm), as detailed in Table 1. By integrating granulometric data (Figure 2 and Table 2) with Mie scattering theory, we demonstrated that Rayleigh and Geometric scattering effects are negligible compared to Undulatory scattering for formazin grains in the 0.38–4.47 μm range. In order to avoid the physical-mathematical difficulties involved in studying this particular scattering theory (see Figures 3 and 4), we have opted for a phenomenological analysis of present turbidity problem. Within this approximate framework, it was possible to introduce the concept of calibration factor γ (see eq 3) and accurately approximate the absorbance curves *A* ≈ *A*_[*R*]_ ([Disp-formula eq6]) by a Padé polynomial as a γ function only (see Figure 5). More specifically, this approximate procedure allowed us to associate the interval between critical values γ_*j*_ (*j* = 1, 2, and 3) with distinct TIR (see eq 8 and Figure 6). In addition, it was also possible to describe these turbidity ranges in terms of a set of incident wavelengths {Λ_*j*_} related to a particular set {*K*_*j*_} of the formazin concentration (see eq 11 and Figure 7).

The proposed methodology combines theoretical and practical domains, offering a robust calibration protocol, and validated for real-world scenarios. By prioritizing empirical data over complex dispersion models, the system achieves simplicity and reliability—essential for industrial and environmental applications such as water quality control and sediment monitoring. The portability of the turbidimeter, combined with adaptive classification (γ), addresses an important limitation of conventional devices: their restricted operating ranges. This presented solution guarantees accuracy even in resource-limited environments, exemplifying its potential for widespread adoption. It is important to recognize the influence of environmental and operational factors on the accuracy of turbidimeter measurements. Fluctuations in ambient temperature can affect the viscosity of the fluid and the kinetics of the particles, thus altering the scattering characteristics of the light. However, for the measurements carried out in the laboratory, the temperature was kept stable, minimizing this effect. The stability of the light source used, a tungsten lamp, is another crucial factor; variations in its intensity can lead to incorrect transmittance readings. To mitigate this, the system was powered by a stabilized power source and an average of 30 measurements was taken. Furthermore, signal transmission via optical fiber can suffer attenuation due to sharp bends or material degradation. To address this, we used a high-quality quartz fiber and kept it in a fixed position to ensure consistent light transmission during the experiments. Although a detailed quantitative analysis of these factors is beyond the scope of this work, which focuses on multiwavelength calibration, we recognize their importance. Validation of the sensor in the field, a natural next step for this research, will require the implementation of strategies to quantify and compensate for the influence of these variables in order to ensure the robustness and accuracy of the measurements in real-world scenarios.

Our future goal is to validate the performance of our prototype in two field scenarios. The first is the process control in the sugar and alcohol industry, specifically monitoring yeast concentration during fermentation for ethanol production. Cell viability and yeast concentration are critical to the efficiency of the process, and the turbidity of the must, which can reach high values, is a direct indicator. The ability of our sensor in operate up to 4000 NTU offers a low-cost alternative for real-time monitoring, optimizing this way fermentation yields. The second use case is in the study of sediment transport in rivers and estuaries, especially during high flow events. In these situations, the concentration of suspended sediment increases dramatically, and the ability to monitor turbidity in real time with a portable and low-cost sensor is crucial for hydrological modeling and environmental impact assessment. Finally, we would like to point out that we are currently trying to improve these approximate results by taking into account possibles random multiple light scattering effects^46,47^ by formazine grains and other sediment particles. Experimental and theoretical efforts in this direction are in progress.

To improve the current results, our future work will focus on modeling the scattering phenomena more explicitly. Initially, we intend to develop a theoretical model based on Mie theory applied to polydisperse suspensions, to more faithfully reflect the size distribution of the formazin particles we have characterized experimentally. The aim will be to check whether a more detailed Mie model can reproduce the results obtained with our phenomenological approach, especially in the undulatory scattering regime, which we have identified as dominant. In addition, we intend to investigate the effects of multiple scattering, which become significant at high turbidity concentrations and which our current model simplifies. Finally, we intend to integrate these refinements into an improved calibration framework, validating it not only with formazine, but also with real sediment samples, thus ensuring greater robustness and applicability of our methodology in complex environmental scenarios.

## Supplementary Material



## Data Availability

The data that support the findings of this study are available throughout the manuscript and in the Supporting Information of this article.

## Notes

Technical drawings of the developed device (ZIP)
