# Supplementary figures and images for: Multi-Wavelength Calibration of a Low-Cost High-Range Turbidimeter: Analysis of the Dispersion Regime
Source: ACS Omega. 2025 Oct 29;10(44):52418–28. doi: 10.1021/acsomega.5c05055 (PMC12612979; doi:10.1021/acsomega.5c05055)

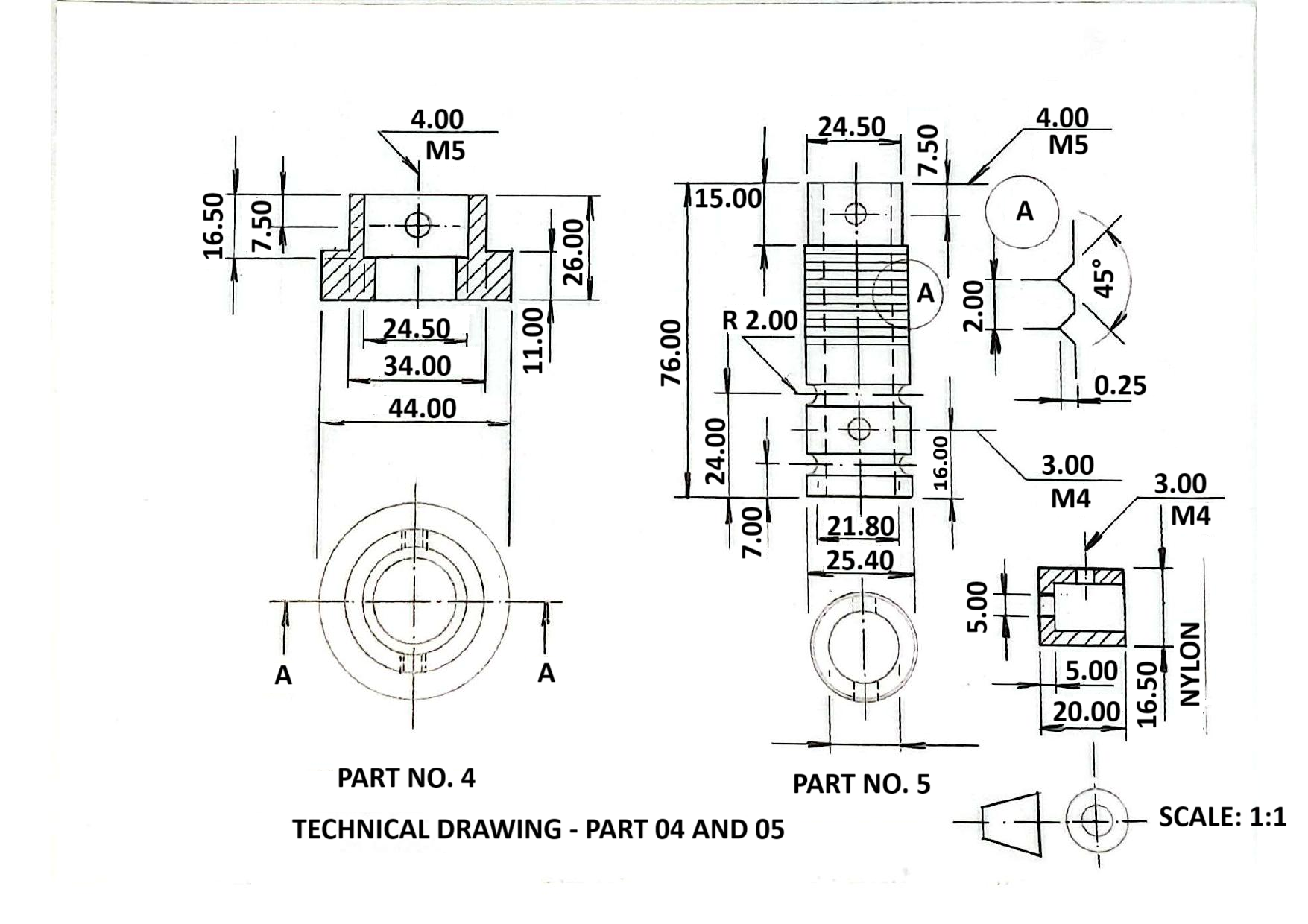

Supplement: Supplementary file 1 [file ao5c05055_si_001.zip › Figures-SM/draw-all_parts.png]

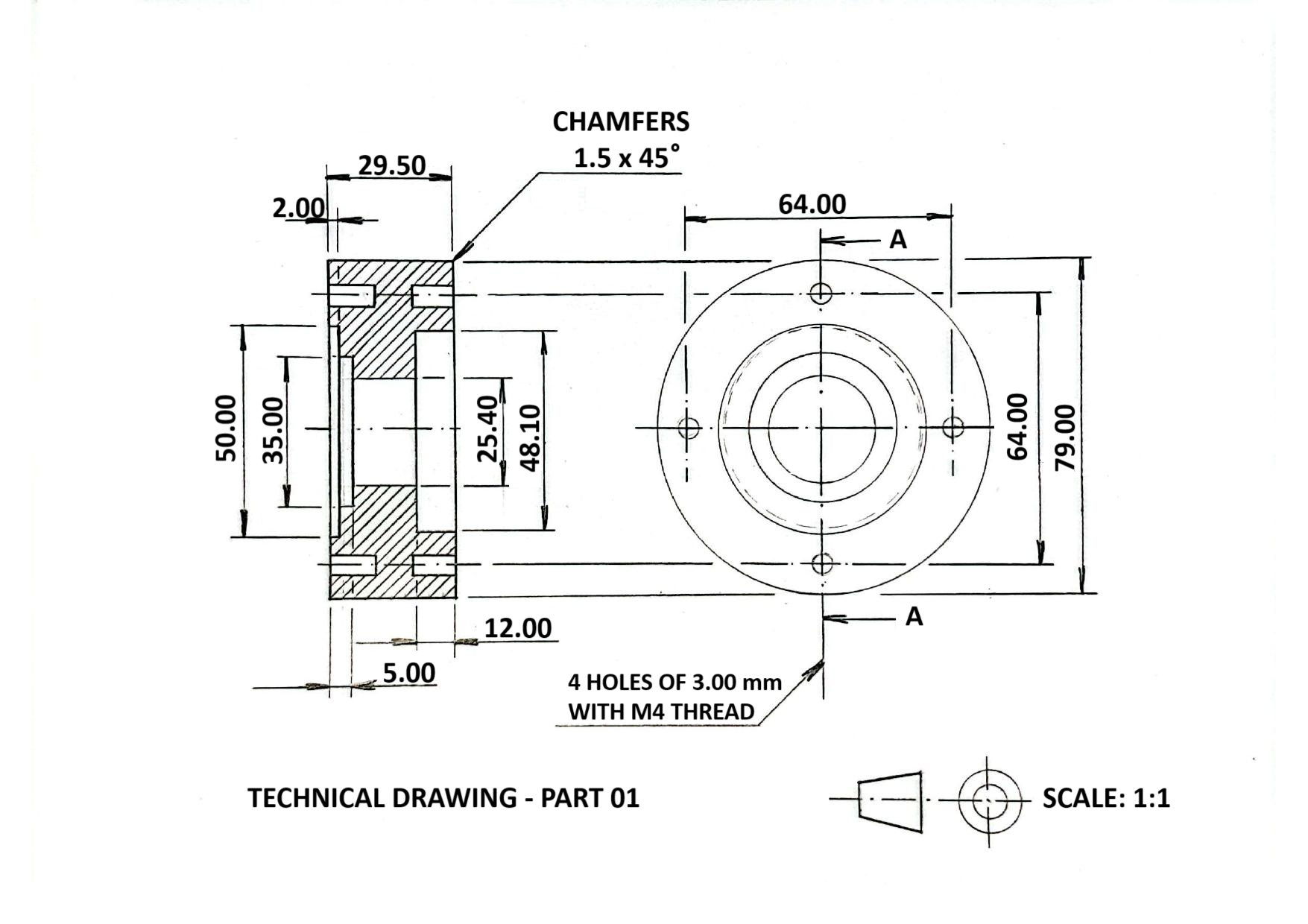

Supplement: Supplementary file 1 [file ao5c05055_si_001.zip › Figures-SM/draw-part1.png]

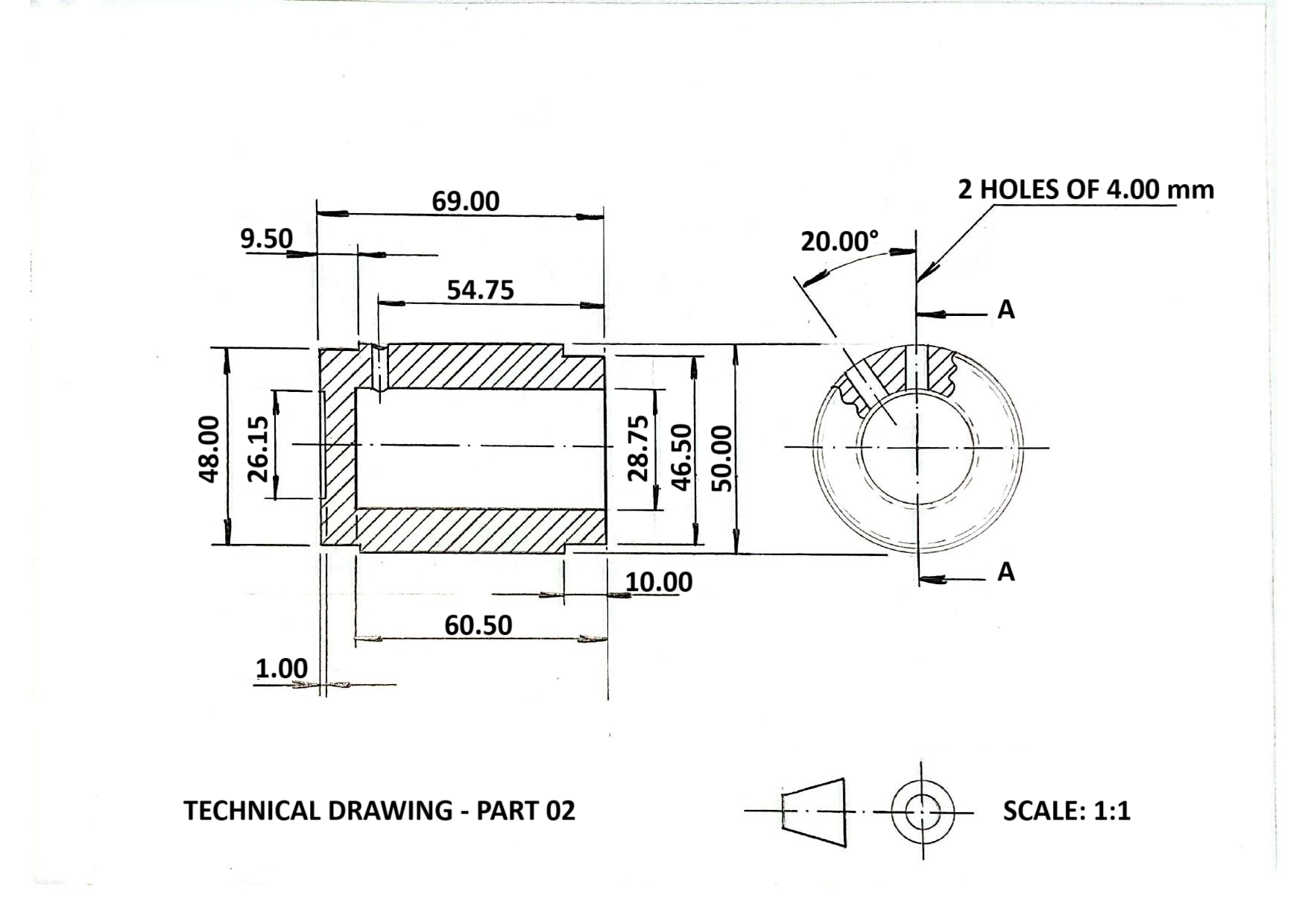

Supplement: Supplementary file 1 [file ao5c05055_si_001.zip › Figures-SM/draw-part2.png]

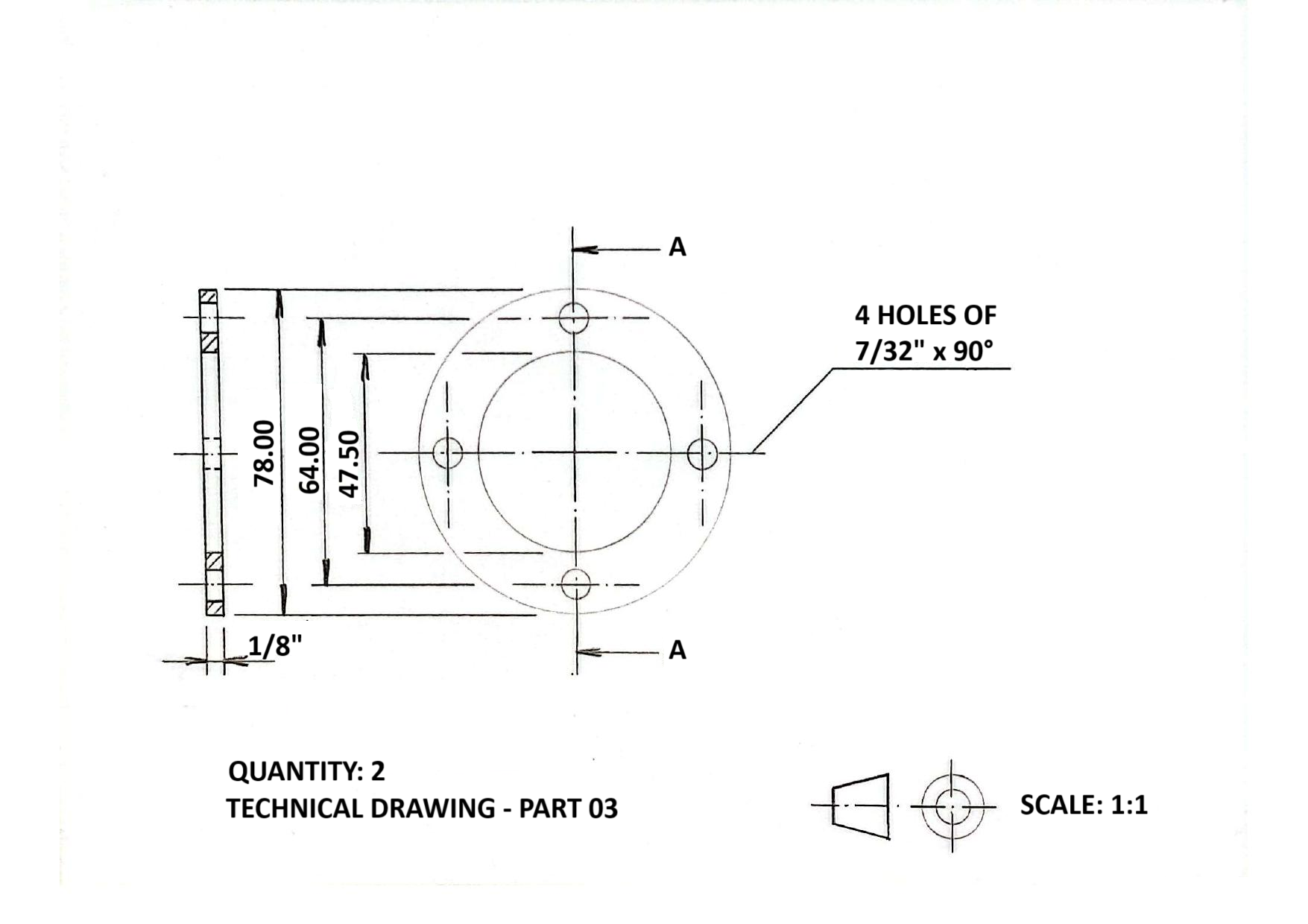

Supplement: Supplementary file 1 [file ao5c05055_si_001.zip › Figures-SM/draw-part3.png]

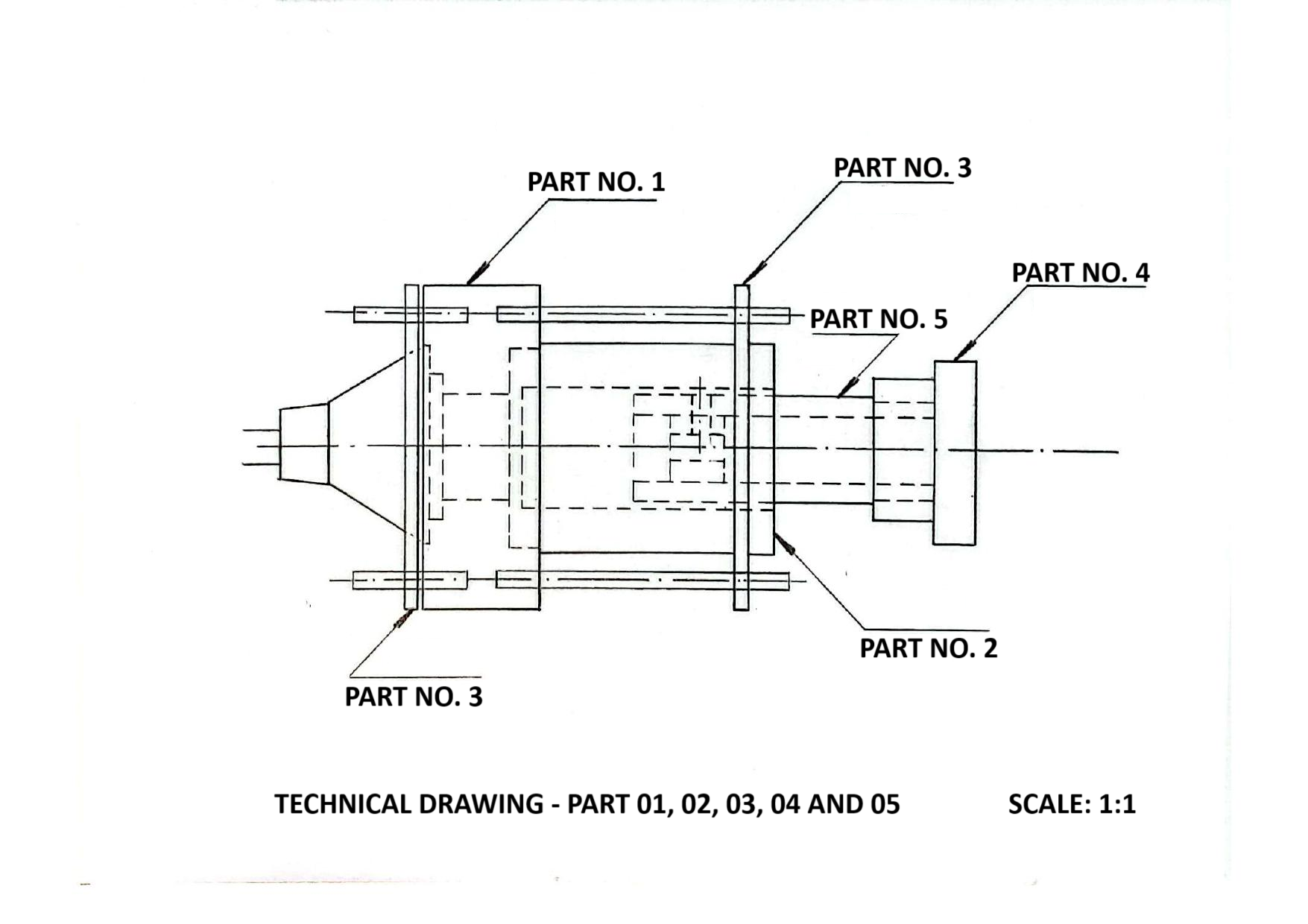

Supplement: Supplementary file 1 [file ao5c05055_si_001.zip › Figures-SM/draw-part4-5.png]
